# Mutual suppression between BHLHE40/BHLHE41 and the MIR301B-MIR130B cluster is involved in epithelial-to-mesenchymal transition of endometrial cancer cells

**DOI:** 10.18632/oncotarget.27061

**Published:** 2019-07-23

**Authors:** Kazuo Asanoma, Emiko Hori, Sachiko Yoshida, Hiroshi Yagi, Ichiro Onoyama, Keisuke Kodama, Masafumi Yasunaga, Tatsuhiro Ohgami, Eisuke Kaneki, Kaoru Okugawa, Hideaki Yahata, Kiyoko Kato

**Affiliations:** ^1^ Department of Obstetrics and Gynecology, Faculty of Medical Sciences, Kyushu University, Fukuoka, Japan

**Keywords:** microRNA, transcription factor, epithelial-to-mesenchymal transition, cell invasion, endometrial cancer

## Abstract

BHLHE40 and BHLHE41 (BHLHE40/41) are basic helix-loop-helix type transcription factors involved in multiple cell activities including epithelial-to-mesenchymal transition (EMT). However, the expression mechanism of BHLHE40/41 in EMT remains unclear. In the present study, we showed that the expression levels of BHLHE40/41 were negatively correlated with those of the microRNA (MIR) 130 family in endometrial cancer (EC) specimens. Our* in vitro* assays indicated that the expression of BHLHE40/41 was suppressed directly by the MIR130 family in a 3’-untranslated region-mediated manner. In EC cells, the MIR130 family promoted EMT and tumor cell invasion by suppressing the expression of BHLHE40/41. We identified the critical promoter region of the *MIR301B*-*MIR130B* cluster for its basal transcription by the transcription factor, SP1. We also found that BHLHE40/41 suppressed the expression of MIR301B and MIR130B, and we identified a binding site in the promoter region for BHLHE40/41. This study is the first to report that BHLHE40/41 and the MIR301B-MIR130B cluster suppressed each other to regulate EMT and invasion of EC cells. We propose that BHLHE40/41 and the MIR130 family are excellent markers to predict the progression of EC cases, and that molecular therapy targeting the MIR130 family-BHLHE40/41 axis may effectively control EC extension.

## INTRODUCTION

Basic helix-loop-helix family member e40 (BHLHE40) and BHLHE41 (BHLHE40/41) are two closely related subfamily members of the basic helix-loop-helix (bHLH) type transcription factors exhibiting more than 90% similarity in the bHLH region, and approximately 40% in total. BHLHE40/41 have been shown to suppress the transcription of their target genes by interacting with GTF2B or TBP, or by recruiting a histone deacetylase at the class B E-box element of the target genes [1–5].

There is increasing evidence showing that BHLHE40/41 play critical roles in cancer development. Tumor suppression by BHLHE40 and/or BHLHE41 has been reported to be mediated by the regulation of cyclins, senescence, epithelial-to-mesenchymal transition (EMT), hypoxia-inducible factors, MAPK1, RELA, or NOTCH1 [6–12]. Several studies, including ours, indicated an inverse correlation between BHLHE40/41 expression levels and clinical stages of EC [8, 12–14]. Furthermore, suppression of EMT by BHLHE40/41 was suggested to be involved in the mechanism [8, 12].

Circadian rhythm factors, hypoxia, numerous growth factors, hormones and cytokines are known to upregulate the expression of BHLHE40/41 [15–17]. However, with regard to the suppression of BHLHE40/41 expression, only a few mechanisms have been reported. Mutual suppression of expression between BHLHE40 and BHLH41 is known [1, 2]. In general, DNA methylation is a well-known mechanism in the suppression of tumor suppressive genes in cancer. However, there are no reports on notable correlations between DNA methylation of *BHLHE40* and cancer [18–20]. A microRNA (miRNA) pathway is another mechanism to regulate gene expression. miRNAs are endogenous small non-coding RNAs of 21–25 nucleotides in length, which regulate the expression of their target genes by mRNA degradation or translational inhibition [21]. Only a few studies reported the expression of BHLHE40 and BHLHE41 was regulated by miRNAs [22–24].

The MIR130 family contains MIR130A, MIR130B, MIR301A, MIR301B, and MIR454, which share a common seed sequence and can target a common sequence. Global expression analysis of the miRNA profile in EC revealed that the MIR130 family is among the upregulated miRNAs in EC compared with normal endometrium [25–27]. In particular, the expression of a MIR130 family member, MIR301B was further upregulated in tissues at clinical stages more than IB compared with those at stage IA [26]. The MIR130 family was also identified among the pan-cancer oncogenic miRNA superfamily [25]. In addition to them, upregulation of the MIR130 family in the process of cancer development has been reported in a variety of cancer types [28–31]. However there are only a few reports on the regulation mechanisms of the MIR130 family in cancer [31–33]. The MIR130 family is known to enhance cell invasion in various types of cancer including EC [28–30, 34, 35]. As onco-miRNAs, MIR130 family members have been reported to target multiple molecules including PPARG, PTEN and TP63 [29, 30, 33–39]. Among the MIR130 family, *MIR301B* and *MIR130B* locate close to each other as a cluster, and their expression levels are suggested to be regulated simultaneously [31].

In the present study, we investigated a novel regulatory mechanism of BHLHE40/41 and MIR130 family expression in EMT of EC cells.

## RESULTS

### Expression pattern of BHLHE40, BHLHE41 and MIR130 family in EC

**Figure 1:**
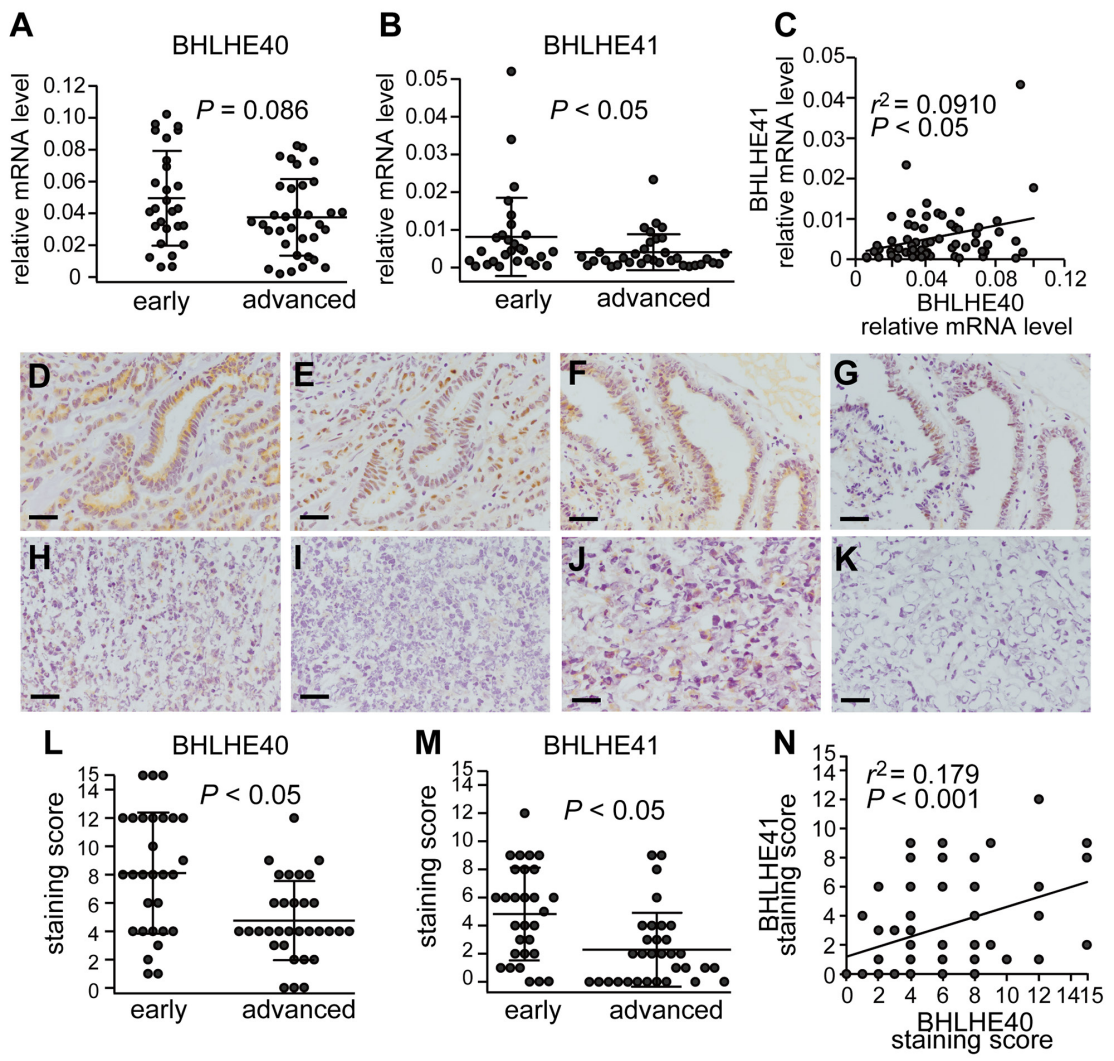
Parallel expression of BHLHE40 and BHLHE41 Sixty-one primary EC specimens were used to analyze the mRNA levels of BHLHE40 **(A)** and BHLHE41 **(B)**. BHLHE40/41 mRNA levels in the EC group at an early stage (stage IA) were compared with those with advanced stages (at or more than stage IB). **(C)** The relationship between BHLHE40 and BHLHE41 mRNA levels from the 61 specimens was analyzed using Pearson’s product-moment correlation coefficient. r-values show correlation coefficients. The 61 EC samples were also analyzed for BHLHE40/41 expression levels by immunohistochemistry. Representative results are shown. A grade 1 endometrioid carcinoma (EAC) case at stage IA **(D, E)**, another grade 2 EAC case at stage IA **(F, G)**, a grade 3 EAC case at stage IB **(H, I)**, and a serous carcinoma case at stage IVB **(J, K)**. Immunohistochemical images with an anti-BHLHE40 antibody (D, F, H, J), and an anti-BHLHE41 antibody (E, G, I, K) are shown. The scale bars indicate 100 µm. The staining scores of immunohistochemical images were analyzed **(L, M)**. The 61 cases were divided into an early stage group and an advanced stage group, as described above. **(N)** The relationship between BHLHE40 and BHLHE41 staining levels from the 61 specimens was analyzed using Pearson’s product-moment correlation coefficient.

In order to study the impact of BHLHE40/41 expression in EC, we first examined their expression levels in EC specimens. Sixty-one cases of surgically removed specimens from primary cancer sites were used for mRNA assays. To determine the correlation between BHLHE40/41 expression and the invasion capacity of cancer, the cases at stage IA were compared with those at or more than stage IB. EC at stage IA indicates cases with no or less than 50% invasion into the adjacent myometrium, and EC at or more than stage IB indicates cases showing more extension into the adjacent uterus, dissemination and/or metastasis. Although there was only a modest difference in the mRNA levels of BHLHE40, the mRNA levels of BHLHE41 were significantly higher in cases at the early stage (stage IA) than in those at advanced stages (at or more than stage IB) (Figure 1A and 1B). A positive correlation was observed between BHLHE40 and BHLHE41 mRNA levels (Figure 1C).

BHLHE40/41 protein levels in the EC specimens were also analyzed by immunohistochemistry. Representative samples shown were positive for BHLHE40/41 (Figure 1D–1G), whereas others were negative (Figure 1H–1K). In contrast to the results obtained from the mRNA assay, BHLHE40/41 staining levels were both higher in cases at the early stage than in those at advanced stages (Figure 1L and 1M). A positive correlation was detected between BHLHE40 and BHLHE41 staining levels (Figure 1N).

**Figure 2:**
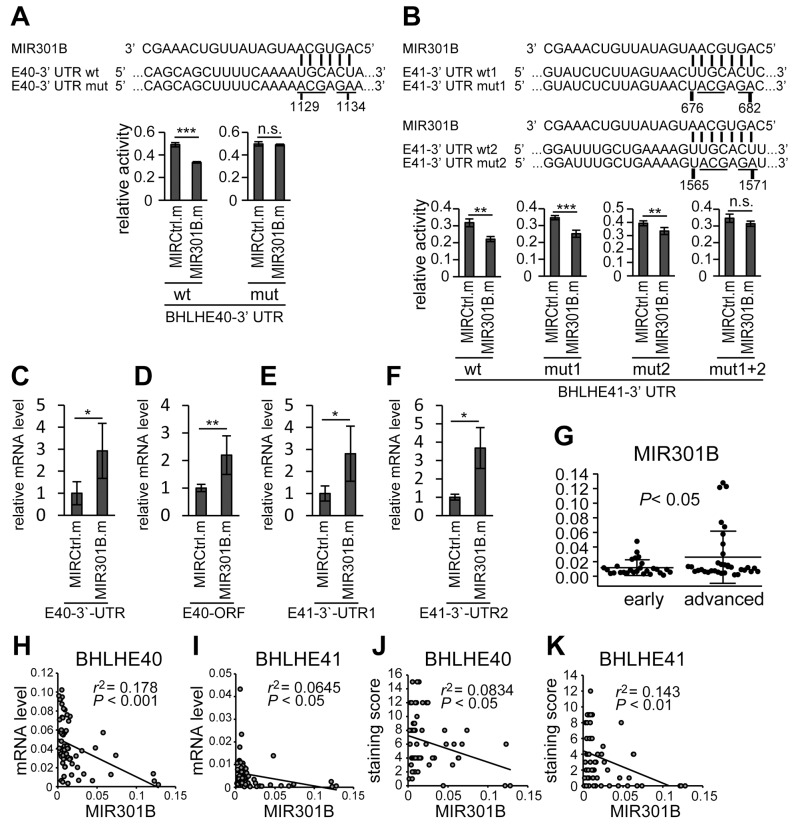
3’-UTRs mediated regulation of BHLHE40/41 expression by MIR301B The reporter activities using 3’-UTRs of BHLHE40 **(A)** and BHLHE41 **(B)** in response to a MIR301B mimic are shown. Data were representative from at least three experiments. Specific association of MIR301B with mRNAs of BHLHE40 **(C, D)** and BHLHE41 **(E, F)** was confirmed by miRNA pulldown assay. The sequence information of the primers to amplify BHLHE40 3’-UTR (C), BHLHE40 ORF region (D), BHLHE41 3’-UTR1 (E), and BHLHE41 3’-UTR2 (F) are shown in Supplementary Table 3. **(G)** The 61 cases divided into the group at the early stage (stage IA) and that at the advanced stages (at or more than stage IB) were analyzed for their expression levels of MIR301B. **(H–K)** Correlations between the expression levels of MIR301B and those of BHLHE40/41 mRNA (H, I) and protein levels (J, K) were analyzed using Pearson’s product-moment correlation coefficient. MIRCtrl.m, control microRNA mimic; MIR301B.m, MIR301B mimic; E40, BHLHE40; E41, BHLHE41, wt, wild type; mut, mutant. A value of *P* < 0.05 was considered significant. n.s., not significant; ^*^, *P* < 0.05; ^**^, *P* < 0.01; ^***^, *P* < 0.001.

The results above suggested that the expression of BHLHE40 and BHLHE41 was regulated by a common mechanism. Then we focused on miRNA-based regulation. Searches for candidate miRNAs targeting both BHLHE40 and BHLHE41 using online prediction algorithms, TargetScan [40], miRDB [41] and TarBase [42], identified the MIR130 family. A reporter assay in HHUA cells using the 3’-UTRs of BHLHE40 and BHLHE41 co-transfected with a mimic of MIR301B, a member of the MIR130 family, showed the suppression of reporter activity (Figure 2A and 2B). Introduction of mutations at candidate targeting sites of the MIR130 family abrogated the suppression (Supplementary Table 1; Figure 2A and 2B). Furthermore, a miRNA pulldown assay showed MIR301B directly associated with mRNA of BHLHE40 and BHLHE41 (Figure 2C–2F). These data suggested that MIR301B directly regulates the expression of BHLHE40/41.

The expression of the MIR130 family was examined in EC samples, which were also used for BHLHE40/41 expression assay. We used TaqMan miRNA assays to detect only mature miRNAs. The expression levels of MIR301A and MIR301B were higher in advanced cases than early cases (Figure 2G; Supplementary Figure 1A–1D). Correlation analysis was performed between the expression levels of MIR130 family members and mRNA and protein levels of BHLHE40/41. There were remarkable correlations observed in several combinations of MIR130 family members and BHLHE40/41, except where these involved MIR454 (Figure 2H–2K; Supplementary Figure 1E–1T). Then MIR454 was excluded from the *in vitro* study thereafter. In particular, there were remarkable correlations in the expression levels between MIR310B and BHLHE40/41 (Figure 2H–2K).

### Forced expression of the MIR130 family in EC cells suppressed the expression of BHLHE40/41 and enhanced cell invasion

**Figure 3:**
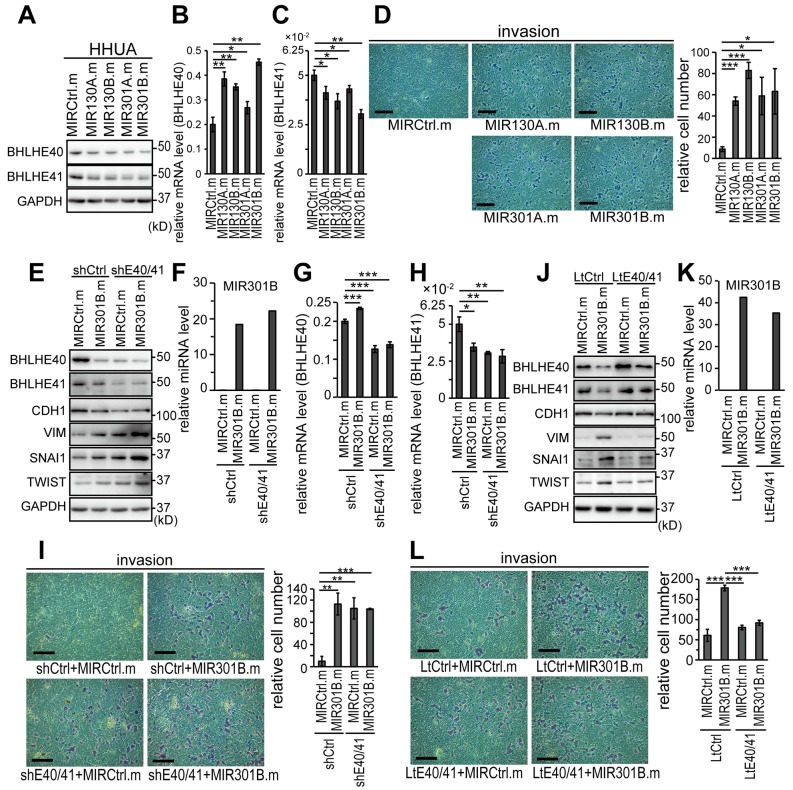
MIR130 family members enhanced EMT and invasion of EC cells by suppressing the protein expression of BHLHE40/41 **(A)** Protein expression of BHLHE40/41 in HHUA cells transfected with mimics of MIR130 family members at a concentration of 25 nM. See also Supplementary Figure 2I and 2J for semi-quantification data. mRNA levels of BHLHE40 **(B)** and BHLHE41 **(C)** in the HHUA cells used in (A). **(D)** *In vitro* cell invasion of the HHUA cells used in (A–C). **(E)** Protein expression of EMT markers in control or BHLHE40/41-knocked-down HHUA cells transfected with a control or MIR301B mimic. **(F)** Expression level of MIR301B in HHUA cells used in (E). mRNA levels of BHLHE40 **(G)** and BHLHE41 **(H)** of HHUA cells used in (E, F). **(I)** *In vitro* cell invasion of HHUA cells used in (E–H). **(J)** Protein expression of EMT markers in control or BHLHE40/41 expressing HHUA cells transfected with a control or MIR301B mimic. **(K)** Expression level of MIR301B in HHUA cells used in (J). **(L)** *In vitro* cell invasion of HHUA cells used in (J, K). (D, I, L) The right graphs showed quantification data of the results. The scale bars indicate 200 µm. Data were representative from three experiments. shE40/41, shBHLHE40+shBHLHE41; LtE40/41, LtBHLHE40+LtBHLHE41; MIRCtrl.m, control microRNA mimic; MIR130A.m, MIR130A mimic; ^*^, *P* < 0.05; ^**^, *P* < 0.01; ^***^, *P* < 0.001.

We first used an EC cell line, HHUA cells, to study the impact of the MIR130 family because HHUA cells are the only EC cell line that abundantly expressed both BHLHE40 and BHLHE41 (Supplementary Figure 2A–2C). Expression of the MIR130 family was also examined in a series of EC cell lines (Supplementary Figure 2D–2G). However there were no correlations between the expression levels of miRNA and BHLHE40/41 (Supplementary Figure 2A–2G). To enforce the expression of MIR130 family members in EC cells, mimics of the microRNA were transfected into HHUA cells. Effective expression of MIR130A, MIR130B, MIR301B and MIR310B was obtained by their mimics transfection (Supplementary Figure 2H). Protein expression of both BHLHE40 and BHLHE41 was suppressed by every mimic of the MIR130 family (Figure 3A; Supplementary Figure 2I and 2J). mRNA expression of BHLHE40 was enhanced by the mimics (Figure 3B). In contrast, the mRNA level of BHLHE41 was suppressed by the mimics (Figure 3C). The forced expression of every MIR130 family member enhanced *in vitro* cell invasion through Matrigel-coated membrane (Figure 3D).

Next, we focused on MIR301B, whose expression level showed the most prominent correlation with those of both BHLHE40 and BHLHE41 (Figure 2H–2K). Dose-dependent expression of MIR301B was observed by transfection of its mimic into HHUA cells (Supplementary Figure 3A). In response to the forced expression of MIR301B, protein expression of BHLHE40 and BHLHE41 was suppressed (Supplementary Figure 3B). Again, mRNA level of BHLHE41 was suppressed and that of BHLHE40 was enhanced (Supplementary Figure 3C and 3D). *In vitro* cell invasion was enhanced by the mimic, and protein analysis showed that an epithelial marker, CDH1, was downregulated, and mesenchymal markers, VIM, SNAI1, SNAI2 and TWIST, were upregulated by the mimic (Supplementary Figure 3B and 3E). These data suggested that the MIR130 family including MIR301B enhanced cell invasion by inducing EMT.

### MIR301B enhanced EMT and cell invasion by suppressing the expression of BHLHE40/41

Our previous study indicated that BHLHE40 and BHLHE41 suppressed EMT and cell invasion by suppressing the transcription of the EMT effectors, TWIST, SNAI1, or SNAI2 [8]. The results above suggested that MIR310B enhanced EMT and cell invasion by inhibiting BHLHE40/41. To evaluate the impact of BHLHE40/41 expression in the EMT and cell invasion induced by MIR310B, we first used HHUA cells, in which both BHLHE40 and BHLHE41 were successfully knocked down (Figure 3E, 3G and 3H). Effective expression of MIR301B was induced by transfection of its mimic (Figure 3F). The enhancement of EMT and *in vitro* cell invasion by MIR301B expression was weakened by knockdown of BHLHE40/41 (Figure 3E and 3I). Next we used HHUA cells stably expressing both BHLHE40 and BHLHE41, which did not contain 3’-UTRs responsive to the MIR130 family (Figure 3J). The enhancement of EMT and cell invasion by MIR301B expression was canceled by forced expression of the BHLHE40/41 unresponsive to MIR301B (Figure 3J–3L).

Furthermore, HEC-1 and HEC-6 cells, which expressed only small amounts of both BHLHE40 and BHLHE41 were used to examine the impact of MIR301B expression. Both BHLHE40 and BHLHE41 were successfully expressed in HEC-1 and HEC-6 cells and their expression suppressed EMT and cell invasion (Supplementary Figure 4A, 4C, 4D and 4F). The forced expression of MIR301B produced only modest changes in both control cells and BHLHE40/41-expressing cells (Supplementary Figure 4A–4F).

### BHLHE41 dominantly regulated the expression of the MIR301B-MIR130B cluster

**Figure 4:**
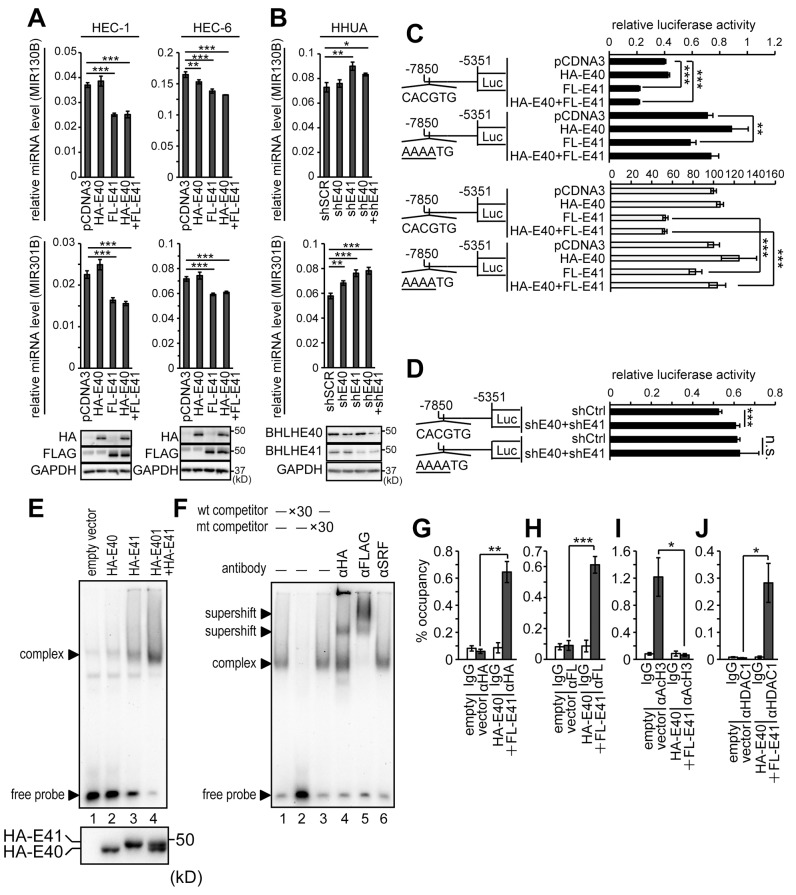
Identification of the BHLHE40/41-responsive site in the promoter of the *MIR301B-MIR130B* cluster **(A)** Expression levels of MIR130B and MIR301B in HEC-1 and HEC-6 cells transfected with vectors to express HA-BHLHE40 and/or FLAG-BHLHE41. The lower panels show the protein expression of HA-BHLHE40 and FLAG-BHLHE41 using anti-HA or -FLAG antibody. **(B)** Expression levels of MIR130B and MIR301B in HHUA cells knocked-down with shBHLHE40 and/or shBHLHE41. The lower panels show the protein expression levels of BHLHE40/41. **(C)** Reporter analysis of the *MIR301B-MIR130B* promoter in HEC-6 cells transfected with HA-BHLHE40 and/or FLAG-BHLHE41. The control activity of the mutant reporter was adjusted to the same value as that of the wild-type reporter to evaluate the effects of BHLHE40/41 expression (C, white bars). **(D)** Reporter analysis of the *MIR301B*-*MIR130B* promoter in HHUA cells knocked-down with both shBHLHE40 and shBHLHE41. **(E,** upper panel**)** EMSA using nuclear extract from 293T cells transfected with HA-BHLHE40 and/or HA-BHLHE41. The nuclear extracts were incubated with labeled E-box2 probe (Supplementary Table 2). **(E,** lower panel**)** Nuclear extracts from 293T cells used for the EMSA were immunoblotted with an anti-HA antibody. **(F)** Nuclear extracts from 293T cells transfected with HA-BHLHE40 and FLAG-BHLHE41 were incubated with labeled E-box2 probe (Supplementary Table 2). Anti-HA or -FLAG antibody was used to form supershifted bands. An anti-SRF antibody was used as a negative control. Chromatin immunoprecipitation assay using 293T cells transfected with HA-BHLHE40 and FLAG-BHLHE41. Protein-DNA complexes immunoprecipitated with each of the anti-HA **(G)**, -FLAG **(H)**, -acetylated Histone H3 **(I)**, and -HDAC1 **(J)** antibodies were used to amplify the E-box by PCR. The 10% input samples were used to calculate the occupancy ratio (%) from the values measured by real-time PCR. Data were representative from at least three experiments. FL, FLAG; E40, BHLHE40; E41, BHLHE41; AcH3, acetylated Histone H3; n.s., not significant; ^*^, *P* < 0.05; ^**^, *P* < 0.01; ^***^, *P* < 0.001.

The results above suggested the impact of the MIR301B-BHLHE40/41 signaling pathway on the EMT and invasion of EC cells. Next, we focused on MIR301B and MIR130B, which are separated by only 245 bp. In general, the distance of a promoter from its miRNA coding region is variable, ranging from a few hundred bases to 20 kb or longer [43]. A perfect canonical E-box (-CACGTG-) was found in the 5’ upstream region of the *MIR301B-MIR130B* in a preliminary search (Supplementary Figure 5A). This discovery led us to study the impact of BHLHE40/41 on the expression of MIR130B and MIR301B. Forced expression of BHLHE40 and/or BHLHE41 in HEC-1 and HEC-6 cells resulted in downregulation of both MIR130B and MIR301B (Figure 4A). This effect is prominent in the case of BHLHE41 expression (Figure 4A). On the other hand, knockdown of BHLHE40 and/or BHLHE41 upregulated both MIR130B and MIR301B in HHUA cells (Figure 4B). A reporter assay was performed using the upstream promoter region (-7850~ -5351 bp from MIR130B) of the *MIR301B-MIR130B* cluster. As expected, the reporter activity was remarkably suppressed by forced expression of BHLHE41 (Figure 4C). On the other hand, knockdown of BHLHE40 and BHLHE41 enhanced the reporter activity (Figure 4D). Introduction of a mutation in the canonical E-box abrogated the effects (Supplementary Table 1; Figure 4C and 4D). There are several E-box sequences in the promoter region of the *MIR301B*-*MIR130B* cluster. An EMSA was performed to examine the affinity of each E-box for BHLHE40/41. The canonical E-box (-CACGTG-) showed prominent affinity to BHLHE40/41 (Supplementary Figure 5B). To examine the affinity of BHLHE40 and BHLHE41 to the canonical E-box, nuclear extracts from 293T cells expressing BHLHE40, BHLHE41, and both BHLHE40 and BHLHE41 were used to form DNA-protein complexes. BHLHE41 showed more abundant complexes than BHLHE40 (Figure 4E, upper panel, compare lanes 2 and 3). We confirmed that these cells expressed a comparable level of BHLHE40 and BHLHE41 (Figure 4E, lower panel). The sequence specific binding of BHLHE40/41 was confirmed by adding of wild type and mutant competitor probes (Supplementary Table 2; Figure 4F). The presence of HA-BHLHE40 and FLAG-BHLHE41 in the complexes was demonstrated by the formation of supershifts after adding of anti-HA or anti-FLAG antibody (Figure 4F, lanes 4 and 5). A ChIP assay was also conducted to show the interaction of BHLHE40/41 and a DNA region containing the canonical E-box (Figure 4G and 4H). Forced expression of BHLHE40/41 resulted in dissociation between acetylated Histone H3 and the DNA region and association between HDAC1 and the DNA region (Figure 4I and 4J) [4, 5].

### SP1 is involved in transcriptional activation of the MIR301B-MIR130B cluster

**Figure 5:**
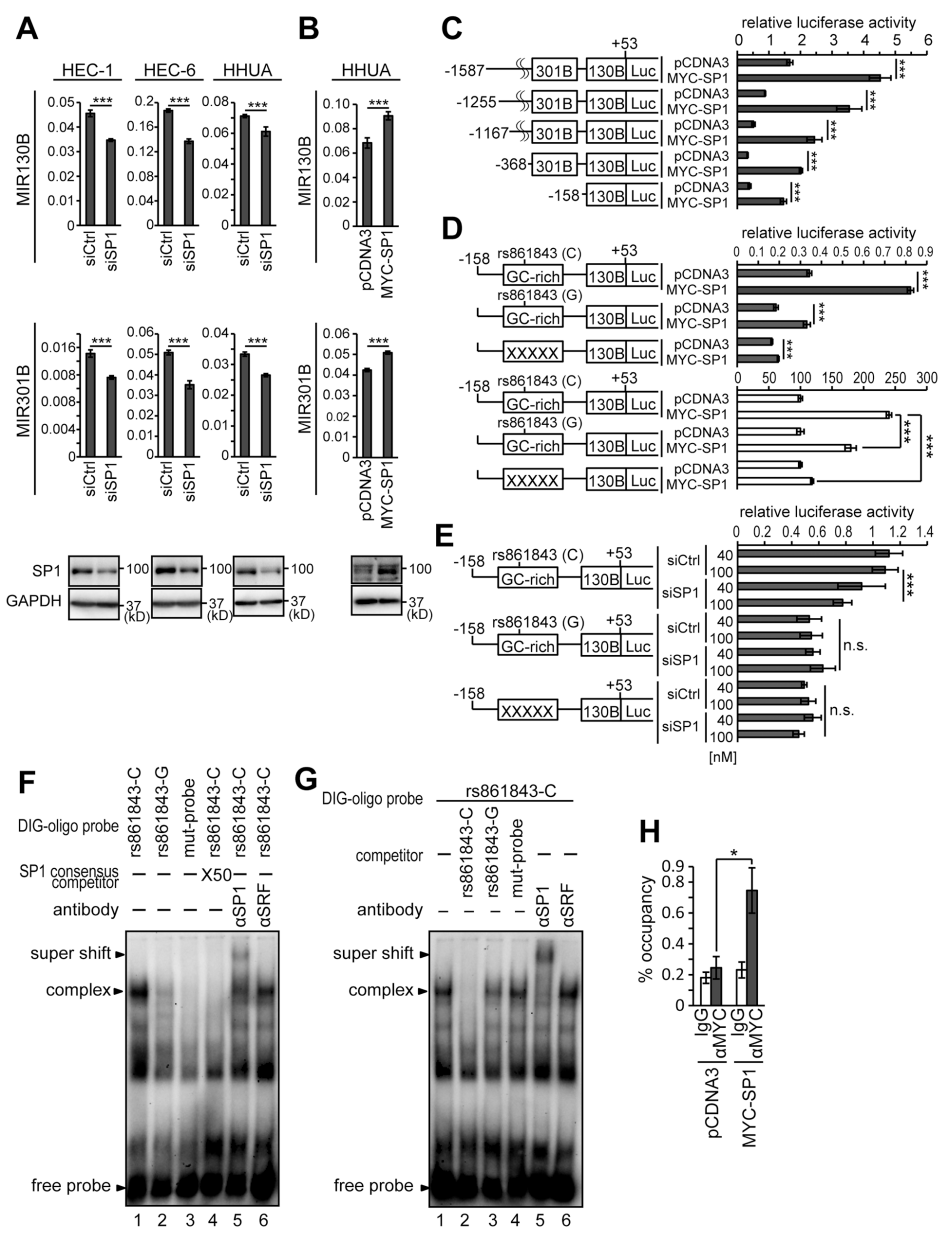
Identification of the SP1-responsive site in the promoter of the *MIR301B-MIR130B* cluster Expression levels of MIR130B and MIR301B in HEC-1, HEC-6 and HHUA cells transfected with siSP1 **(A)** or a vector to express MYC-SP1 **(B)**. The lower panels show the protein expression of SP1 using an anti-SP1 antibody (A, B). **(C)** Five kinds of reporters possessing upstream regions of -1587, -1255, -1167, -368 and -158 from the transcription start site of *MIR130B* were analyzed for their activity. **(D)** Reporter analysis of the *MIR301B*-*MIR130B* promoter in HHUA cells transfected with MYC-SP1. The control activity of the mutant reporter was adjusted to the same value as that of the wild type reporter to evaluate the effects of BHLHE40/41 expression **(D,** white bars**)**. **(E)** Reporter analysis of the *MIR301B*-*MIR130B* promoter in HHUA cells transfected with siSP1 at a concentration of 40 or 100 nM. **(F)** EMSA using nuclear extract from 293T cells incubated with various types of labeled SP1 binding site (SP1BS) probes. Anti-SP1 antibody was used to form a supershifted band. Anti-SRF antibody was used as a negative control. **(G)** Nuclear extract from 293T cells was incubated with the labeled SP1BS and various types of competitor probes were used. **(H)** ChIP assay using 293T cells transfected with MYC-SP1. Protein-DNA complexes immunoprecipitated with an anti-MYC antibody were used to amplify SP1BS by PCR. The 10% input samples were used to calculate the occupancy ratio (%) from the values measured by real-time PCR. Data were representative from at least three experiments. n.s., not significant; ^*^, *P* < 0.05; ^***^, *P* < 0.001.

Using the preliminary search of the promoter region of the *MIR310B*-*MIR130B* cluster with rVista 2.0 (https://rvista.dcode.org/), several candidate binding sites for a transcription factor, SP1, were identified. We first knocked-down the expression of SP1 by siRNA (siSP1) transfection in HEC-1, HEC-6, and HHUA cells. Successful knockdown of SP1 in HEC-1, HEC-6, and HHUA cells resulted in downregulation of both MIR130B and MIR301B (Figure 5A). On the other hand, forced expression of SP1 in HHUA cells, which originally expressed relatively low levels of SP1 resulted in upregulation of both MIR130B and MIR301B (Figure 5B). Next, we generated a reporter containing a proximal promoter region (-1587~ +53 bp from *MIR130B*: pMIR130B-1587), which is relatively conserved among human and mouse. To narrow down the region responsible for SP1 binding, 4 kinds of truncated reporters along with pMIR130B-1587 reporter were used for reporter assay to examine the effect of SP1 expression. Similar to pMIR130B-1587 reporter, the shortest reporter, pMIR130B-158 still showed upregulation of the reporter activity by forced expression of SP1 (Figure 5C). A search of -158~ +53 region by rVista 2.0 identified a GC-rich region, a candidate site for SP1 binding. Introduction of mutations in the GC-rich region abrogated the upregulation of reporter activity in response to forced expression of SP1 (Supplementary Table 1; Figure 5D). As expected, knockdown of SP1 expression suppressed the reporter activity (Figure 5E). In contrast, siSP1 failed to suppress the activity of a mutant reporter (Supplementary Table 1; Figure 5E).

A search for a single nucleotide polymorphism (SNP) in the NCBI database (https://www.ncbi.nlm.nih.gov/projects/SNP/) identified an SNP, rs861843 (C/G), in the GC-rich region. A reporter with rs861843-G showed less activation by SP1 than that with rs861843-C (Figure 5D). Similar to the mutant reporter, the reporter with rs861843-G showed resistance to SP1 knockdown (Figure 5E). Affinity between the GC-rich region and SP1 was assayed using an EMSA. The GC-rich probe with rs861843-C had a higher affinity with SP1 compared with that with rs861843-G or mutations (Supplementary Table 2; Figure 5F and 5G). The presence of SP1 in the DNA-protein complex was demonstrated by supershift formation after addition of the anti-SP1 antibody (Figure 5F and 5G). SP1 binding to the GC-rich region was also confirmed using ChIP assay in 293T cells (Figure 5H). HEC-1, HEC-6, HHUA, and 293T cells all have C/C alleles at rs861843. To study the impact of rs861843 in EC prevalence and EC development, blood samples from 300 EC cases and 150 age-matched healthy controls were examined for their genotypes at rs861843. All the samples were from Japanese people. Unexpectedly, all the genomic samples had C/C at rs861843.

### Inhibition of MIR130B and MIR301B resulted in upregulation of BHLHE40/41 expression

**Figure 6:**
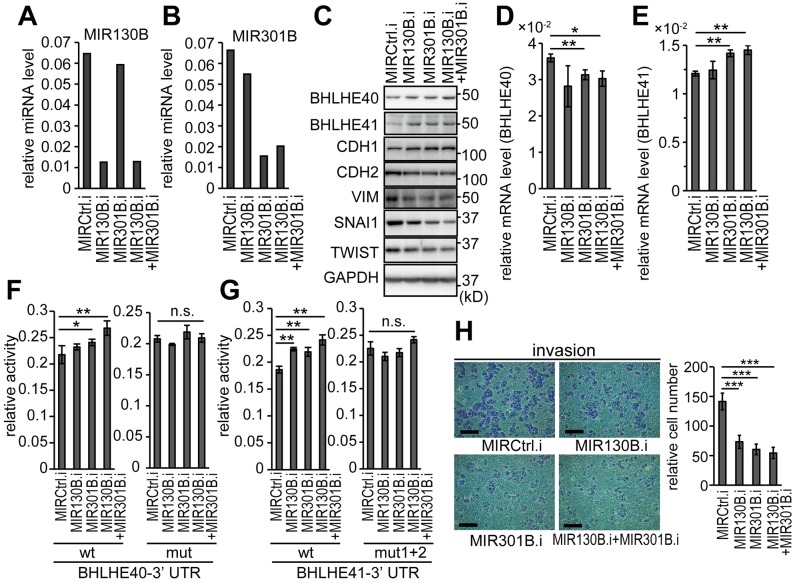
Inhibition of MIR130B and MIR301B enhanced the protein expression of BHLH40/41 and suppressed EMT in EC cells Expression levels of MIR130B **(A)** and MIR301B **(B)** in HEC-6 cells transfected with their inhibitors at a concentration of 50 nM. **(C)** Protein expression of BHLHE40/41 and EMT markers in HEC-6 cells used in (A, B). mRNA levels of BHLHE40 **(D)** and BHLHE41 **(E)** in HEC-6 cells used in (A–C). The reporter activities using 3’-UTRs of BHLHE40 **(F)** and BHLHE41 **(G)** in response to inhibitors of MIR130B and MIR301B are shown. The left graphs show the results from wild-type reporters and the right graphs show the results from mutant reporters (F, G). **(H)** *In vitro* cell invasion of HEC-6 cells used in (A–E). The right graph shows quantified results data (H). Data were representative from at least three experiments. The scale bars indicate 200 µm. MIRCtrl.i, control microRNA inhibitor; MIR130B.i, MIR130B inhibitor; MIR301B.i, MIR301B inhibitor; n.s., not significant; ^*^, *P* < 0.05; ^**^, *P* < 0.01; ^***^, *P* < 0.001.

The impact of inhibition of MIR130B and MIR301B was examined in HEC-6 cells, which expressed only low levels of BHLHE40/41. Successful suppression of MIR130B and MIR301B expression in each cell line was obtained by transfection of inhibitors for MIR130B and MIR301B (Figure 6A and 6B). Inhibition of MIR130B and MIR301B upregulated the protein levels of both BHLHE40 and BHLHE41 (Figure 6C). In contrast to the observation with the MIR130B and MIR301B mimics, their inhibitors suppressed the mRNA levels of BHLHE40 but enhanced mRNA levels of BHLHE41 (Figure 6D and 6E; also see Figure 3B and 3C). Reporter activity of 3’-UTRs of BHLHE40 and BHLHE41 was enhanced by inhibitors of MIR130B and MIR301B (Figure 6F and 6G). Inhibition of MIR130B and MIR301B suppressed EMT and *in vitro* cell invasion (Figure 6C and 6H). Throughout the assays using inhibitors, simultaneous transfection of both MIR130B and MIR301B inhibitors led to remarkable results. Similar results were obtained using HEC-1 cells (Supplementary Figure 6).

## DISCUSSION

BHLHE40/41 are known to be involved in carcinogenesis, cancer development, invasion, and metastasis. In our previous study, we showed that BHLHE40/41 inhibited EMT and cell invasion of EC cells by suppressing the transcription of SNAI1, SNAI2 and TWIST1 [8]. As suggested tumor suppressors, the expression of BHLHE40/41 was downregulated in cancer cases at advanced stages (Figure 1B, 1L and 1M). A correlation analysis showed that the expression levels of BHLHE40 and BHLHE41 were positively correlated (Figure 1C and 1N). Based on the results, we assumed that the expression of both BHLHE40 and BHLHE41 were regulated by a common unknown mechanism. We identified target sequences for the MIR130 family in 3’-UTRs of both BHLHE40 and BHLHE41 (Figure 2A and 2B). A reporter assay demonstrated that MIR301B suppressed the reporter activity in a sequence-specific manner (Figure 2A and 2B). Although there were two candidate sites for the MIR130 family in 3’-UTR of BHLHE41, the distant site (+1565~ +1571) was dominant in suppression of the reporter activity (Figure 2B).

An *in vitro* assay indicated that mimics of MIR130 family members suppressed the protein expression of BHLHE40/41 in EC cells (Figure 3A; Supplementary Figure 3B). As expected, MIR130 family members suppressed the mRNA expression of BHLHE41 (Figure 3C; Supplementary Figure 3D). However, the MIR130 family upregulated the mRNA level of BHLHE40 (Figure 3B; Supplementary Figure 3C). In contrast to the case of mimic transfection, inhibitors of MIR130B and MIR301B upregulated BHLHE41 and downregulated BHLHE40 on the mRNA levels (Figure 6D and 6E; Supplementary Figure 6D and 6E). These results suggested that the MIR130 family suppressed the protein expression of BHLHE41 by an mRNA degradation mechanism. In contrast, the MIR130 family was suggested to suppress the protein expression of BHLHE40 by a mechanism other than mRNA degradation, such as translational repression [21]. Because mutual suppression of expression between BHLHE40 and BHLH41 is known [1, 2], it might be assumed that the suppression of BHLHE41 protein by forced expression of the MIR130 family resulted in enhanced transcription of BHLHE40.

Expression analysis using clinical samples showed that miRNA levels of MIR130 family members had negative correlations with mRNA and protein levels of BHLHE40/41 (Figure 2H–2K; Supplementary Figure 1E–1T). In particular, the MIR301B levels had prominent correlations with all of the mRNA and protein levels of BHLHE40 and BHLHE41 (Figure 2H–2K). These results were contradictory to the data obtained from the *in vitro* assay because MIR130 family mimics upregulated BHLHE40 mRNA levels in HHUA cells (Figure 3B; Supplementary Figure 3C). The precise mechanism of this contradiction remains to be dissolved. The regulation of MIR301B-MIR130B and BHLHE40/41 is not one-way pathway. As shown in this study, there is a mutual regulation mechanism between MIR301B-MIR130B and BHLHE40/41. The increased mRNA levels of BHLHE40 in response to MIR301B-MIR130B may in turn suppress MIR301B-MIR130B expression by upregulation of BHLHE40 translation. To support the complexity of regulation between BHLHE40 mRNA and protein, analysis from the clinical sample showed that there was no correlation between mRNA level and protein level of BHLHE40 (Supplementary Figure 7A). In contrast, BHLHE41 had a remarkable positive correlation between mRNA and protein levels (Supplementary Figure 7B).

*MIR130B* and *MIR301B* are very close subfamily members, which form a cluster and seem to share a promoter [31]. We focused on this cluster because the MIR301B expression had a remarkable correlation with the clinical signature and expression levels of both MIR130B and MIR301B showed a negative correlation with those of BHLHE40 and BHLHE41 (Figure 2H–2K; Figure 1E–1T). Inhibition of MIR130B and MIR301B resulted in upregulation of BHLHE40/41 in HEC-1 and HEC-6 cells (Figure 6C; Supplementary Figure 6C). A reporter assay and expression analysis of BHLHE40/41, and an *in vitro* invasion assay showed that inhibition of MIR301B had stronger effects than that of MIR130B and inhibition of both MIR130B and MIR301B had the most significant effects (Figure 6C–6H; Supplementary Figure 6C–6H). It can be presumed that because each EC cell line expressed comparable levels of MIR130 family members, multiple inhibitions of the members had stronger effects (Supplementary Figure 2D–2G).

We also focused on the regulatory mechanism of the MIR301B-MIR130B cluster. There are only a couple of studies reporting the regulation of MIR301B-MIR130B expression [31–33]. Because a perfect canonical E-box (-CACGTG-) was found in the 5’ upstream region of the *MIR301B*-*MIR130B* cluster, we presumed that mutual inhibition between MIR301B-MIR130B and BHLHE40/41 might be involved in EC development. As expected, BHLHE40/41 suppressed the expression of MIR130B and MIR301B (Figure 4A and 4B). Compared with BHLHE40, BHLHE41 had stronger effects on the expression of MIR130B and MIR310B (Figure 4A and 4B). This is also the case with reporter activity (Figure 4C). EMSA showed that BHLHE41 had higher affinity for the E-box than BHLHE40 (Figure 4E, compare lane 2 with lane 3). By adding an anti-HA antibody to the protein-DNA complex, only a small part of the complexes were supershifted (Figure 4F, lane 4). In contrast, adding an anti-FLAG antibody supershifted all the complexes (Figure 4F, lane 5). These results also suggested that BHLHE41 had stronger affinity to the E-box. BHLHE40 and BHLHE41 are known to form a homodimer or a heterodimer to function as transcription factors [44, 45]. Previously, our immunoprecipitation assay showed that the BHLHE41-BHLHE41 homodimer was the preferred form to the BHLHE40-BHLHE40 homodimer or the BHLHE40-BHLHE41 heterodimer [8]. This evidence also supports why BHLHE41 had stronger effects compared with BHLHE40 (Figure 4A–4C). Consistent with our data, Hamaguchi et al. also showed that BHLHE41 had stronger effects compared with BHLHE40 [46].

In this study, we identified a SNP, rs861843 in the GC-rich SP1 binding site of the *MIR301B*-*MIR130B* cluster. The C/G polymorphism at rs861843 significantly affected the reporter activity and the affinity of SP1 for the SP1 binding site (Figure 5D–5G). The frequency of the minor allele, G, varies depending on race. African people have a G allele at a frequency of 0.199. In contrast, East Asian people have a G allele at a frequency of 0.001 (NCBI, dbSNP, https://www.ncbi.nlm.nih.gov/snp/rs861843). Our present assay based on Japanese samples from 300 EC cases and 150 healthy controls showed that all the samples had C/C genotypes at rs861843. Rs861843 could have some impact in African and European people, who have high frequency of a G allele, on EC prevalence or EC development.

In conclusion, we identified the MIR130 family as negative regulators of BHLHE40/41 expression in EC cells. We also identified a critical regulation of MIR301B and MIR130B expression by the transcription factor SP1 and BHLHE40/41. This study is the first to report a novel mutual regulation among BHLHE40/41 and miRNAs in cancer cells. Our results suggest that BHLHE40/41 and the MIR301B-MIR130B cluster suppressed each other to regulate EMT and cell invasion in EC. We propose that BHLHE40/41 and the MIR130 family are excellent markers to predict the progression of EC cases, and that molecular therapy targeting the MIR130 family-BHLHE40/41 axis may effectively control EC extension.

## MATERIALS AND METHODS

### Cell lines

HEC-1, HEC-6, HHUA, Ishikawa and 293T cells were obtained and grown as described previously [8]. KLE and AN3 CA cells were from the American Type Culture Collection (ATCC) (Manassas, VA, USA). 293T, KLE, AN3CA and Ishikawa cells were used within 6 months of receipt. The identities of HEC-1, HEC-6, and HHUA cells were confirmed by the Japanese Collection of Research Bioresources (JCRB) cell bank using DNA profiling (short tandem repeat).

### Patients and tissue samples

Sixty-one EC patients who underwent surgery at the Department of Obstetrics and Gynecology of Kyushu University Hospital between 2005 and 2010 were recruited for this study. The 61 EC primary specimens (29 cases at stage IA, 15 at stage IB, 1 at stage IIA, 1 at stage IIB, 2 at stage IIIB, 11 at stage IIIC, and 2 at stage IVB based on surgical staging of FIGO 2008; 56 endometrioid carcinoma cases including 26 at grade 1, 20 at grade 2, and 10 at grade 3, and 5 serous carcinoma) were used in mRNA, miRNA assays and immunohistochemistry. All patients involved in this study provided their written informed consent. This study was approved by the Ethical Committee of Kyushu University.

### Reverse transcription (RT)-qPCR and TaqMan miRNA assay

Total RNA including miRNA from tissue samples and cultured cells was extracted using a mirVana miRNA Isolation Kit (Thermo Fisher Scientific, Waltham MA, USA). RT-qPCR was performed as described previously [8, 47]. The sequence information of the primers used is shown in Supplementary Table 3. TaqMan qPCR was performed using TaqMan MicroRNA Reverse Transcription Kit and TaqMan Fast Advanced Master Mix. (Applied Biosystems, Foster City, CA, USA). The relative expression levels of target genes were calculated after normalization using those of SNORD44. TaqMan Assay Name: RNU44 for SNORD44; has-miR-130a-3p for MIR130A; has-miR-130b-3p for MIR130B; has-miR-301a-3p for MIR301A; has-miR-301a-3p for MIR301B; has-miR454-3p for MIR454 (Applied Biosystems).

### Immunohistochemistry

Immunohistochemistry was performed as described previously using antibodies as follows: anti-BHLHE40 (HPA028921, Atlas Antibodies, Stockholm, Sweden) or anti-BHLHE41 (E-4, Santa Cruz Biotechnology, Santa Cruz, CA, USA) antibody [8]. The nuclear expression of BHLHE40/41 was evaluated using a staining scoring system modified from that described by Allred et al. [48]. Staining scores were calculated by multiplying the proportion score by the intensity score.

### Immunoblotting

Immunoblotting was performed as described previously using primary antibodies as follows: anti-BHLHE40 (S-8), -SNAI1 (H-130), -SNAI2 (D-19), -SP1 (PEP2), -TWIST1 (H-81), -VIM (V-9), -FN1 (EP5), -CDH1 (H-108), -CDH2 (H-63), and -GAPDH (FL-335) (Santa Cruz Biotechnology). Anti-BHLHE41 (S8568), -HA (HA-7), and -FLAG (M5) antibodies were from Sigma-Aldrich (St. Louis, MO, USA) [8, 47].

### Plasmid transfection, lentivirus vector transduction and luciferase assay

The pCDNA3 vectors to express HA- or FLAG-tagged human BHLHE40 and BHLHE41, and MYC-tagged human SP1, the lentivirus vectors to express HA-tagged BHLHE40 and FLAG-tagged BHLHE41, the lentivirus vectors to knockdown BHLHE40 and BHLHE41 were prepared as described previously [8].

Several DNA regions upstream of *MIR130B* (spanning -7850 bp to -5351 bp, and -1587, -1255, -1167, -368 and -158 bp to +53 bp from the transcription start site) were amplified by PCR and ligated into a pGL4.22-basic luciferase vector (Promega). The 3’- UTRs of BHLHE40 and BHLHE41 (spanning +12 bp to +1545 bp of 3’-UTR for BHLHE40 and +14 bp to +1996 bp of 3’-UTR for BHLHE41) were amplified by PCR and ligated into the pmirGLO Dual-Luciferase miRNA Target Expression Vector (Promega). The sequences of the forward primers to generate the mutants are shown in Supplementary Table 1. The DNA sequence of each construct was confirmed by sequencing reactions. In reporter assays, cells (1×10^5^) were transfected with 200 ng of each luciferase reporter, 100 ng of an expressing vector or 50 nM of miRNA mimic/inhibitor, and 5 ng of pRL-TK vector (Promega) for a pGL4.22-luciferase vector using Lipofectamine 3000 reagent (Invitrogen). Twenty-four hours after transfection, cell lysates were collected and assayed using a Dual-Luciferase Reporter Assay System kit (Promega). Firefly luciferase activity values were normalized using those of Renilla luciferase activity.

### siRNA and miRNA mimics/inhibitors transfection

Double-stranded small interfering RNA (siRNA) for SP1 (siSP1) (sc-29487) was purchased with control siRNA (sc-37007) from Santa Cruz Biotechnology. Mimics of MIR130A, MIR130B, MIR301A, MIR301B, and inhibitors for MIR130B and MIR301B were purchased with a control miRNA mimic and inhibitor from Dharmacon (miRIDIAN microRNA mimics and inhibitors, Dharmacon, Lafayette, CO, USA). siSP1 and miRNAs mimics/inhibitors were transfected into cells using Lipofectamine 3000 reagent (Invitrogen) at a final concentration of 40 or 100 nM for siSP1 and 25 or 50 nM for miRNAs mimics/inhibitors, respectively.

### miRNA pulldown assay

HHUA cells transfected with 3’-biotinylated negative control miRNA mimic or 3’-biotinylated MIR301B mimic (Qiagen, Hilden, Germany) were used for miRNA pulldown assay as described previously [49]. Precipitated RNA samples using streptavidin-coated magnetic beads (GE Healthcare Life Sciences, Chicago, IL, USA) were reverse-transcribed and used for qPCR [8, 47]. The sequence information of the primers used is shown in Supplementary Table 3.

### Electrophoretic mobility shift assay

Electrophoretic mobility shift assays (EMSAs) were performed using nuclear extracts from 293T cells expressing HA-BHLHE40 and/or HA- or FLAG-BHLHE41 as described previously [8]. The sequences of the probes used for BHLHE40/41 and SP1 binding are shown in Supplementary Table 2. For supershift formation, anti-HA, -FLAG (Sigma-Aldrich), -SP1, or -SRF (Santa Cruz Biotechnology) antibody was added to the incubation mixture.

### Chromatin immunoprecipitation assay

Chromatin immunoprecipitation (ChIP) assays were performed as described previously [8, 50]. The DNA-protein complex was immunoprecipitated using anti-HA (ab9110, Abcam), -FLAG (M5, Sigma-Aldrich), -acetylated Histone H3 (Millipore), -HDAC1 (ab7028, Abcam) antibodies. Precipitated DNA samples were used to amplify the E-box and SP1 binding site (SP1BS) of the *MIR301B-130B* promoter with the primers shown in Supplementary Table 3.

### Transwell chamber assay

Cell invasion were evaluated using a transwell chamber assay as described previously [8, 47]. A total of 5.0×10^4^ cells were plated in the upper wells without serum, separated by Matrigel-coated membrane. Complete growth medium with 10% fetal bovine serum was placed in the lower wells. After 24 hours for HEC-1 and after 48 hours for HHUA cells and HEC-6 cells, the membranes were collected for analysis.

### Blood samples and SNP genotyping analysis

Blood samples from 300 EC cases and 150 age-matched healthy controls were examined for their genotypes at rs861843. This study is a part of the cohort study approved by the Ethical Committee of Aichi Cancer Center. SNP genotyping analysis at rs861843 was performed using TaqMan SNP Genotyping Assay (Assay ID: C___8740703_20, Applied Biosystems). The data was processed using TaqMan Genotyper Software (Applied Biosystems).

### Statistics

Data are represented as the mean ± standard deviation (SD). Case-control data were analyzed using the Mann–Whitney U test. The correlation analysis was performed using Pearson’s product-moment correlation coefficient. The significance of these relationships was determined using the *F*-test. Reporter assay and qPCR assay data were analyzed with two-sided Student’s *t*-test. Welch’s test was applied when heteroscedasticity was suspected. *F*-test was used to test if a give set of data had the similar variance. A value of *P* < 0.05 was considered significant.

## SUPPLEMENTARY MATERIALS FIGURES AND TABLES


