# Supplementary material for: Mutual suppression between BHLHE40/BHLHE41 and the MIR301B-MIR130B cluster is involved in epithelial-to-mesenchymal transition of endometrial cancer cells
Source: Oncotarget. 2019 Jul 23;10(45):4640–54. doi: 10.18632/oncotarget.27061 (PMC6659797; doi:10.18632/oncotarget.27061)
Supplement: Supplementary file 1 [file oncotarget-10-4640-s001.pdf]

Mutual suppression between BHLHE40/BHLHE41 and the MIR301B-MIR130B cluster is involved in epithelial-to-mesenchymal transition of endometrial cancer cells

SUPPLEMENTARY MATERIALS

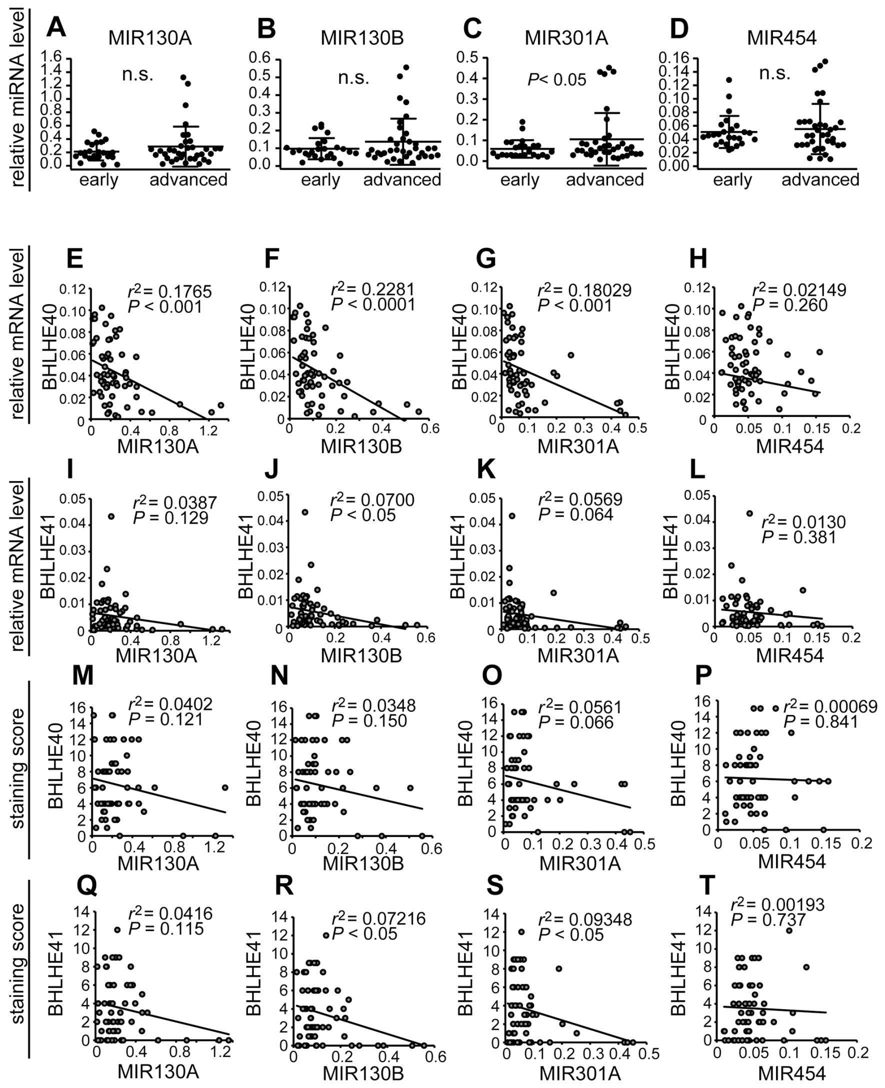

Supplementary Figure 1: The expression of MIR130 family members and their correlations with BHLHE40/41 levels in endometrial cancer specimens. Surgical samples from the 61 EC patients were analyzed for expression levels of MIR130 family members using TaqMan assays. The 61 cases divided into the group at the early stage (stage IA) and that at the advanced stages (at or more than stage IB) were analyzed for their expression levels of MIR130A **(A)**, MIR130B **(B)**, MIR301A **(C)**, MIR454 **(D)**. Correlations between the expression levels of MIR130 family members and those of BHLHE40/41 mRNA and protein levels were analyzed using Pearson's product-moment correlation coefficient. r-values show correlation coefficients. Correlations between expression levels of MIR130 family members and each of mRNA level of BHLHE40 **(E–H)**, mRNA level of BHLHE41 **(I–L)**, protein level of BHLHE40 **(M–P)** and protein level of BHLHE41 **(Q–T)** were analyzed. For MIR130 family members, MIR130A (E, I, M, Q), MIR130B (F, J, N, R), MIR301A (G, K, O, S), MIR454 (H, L, P, T) were analyzed. A value of $P < 0.05$ was considered significant.

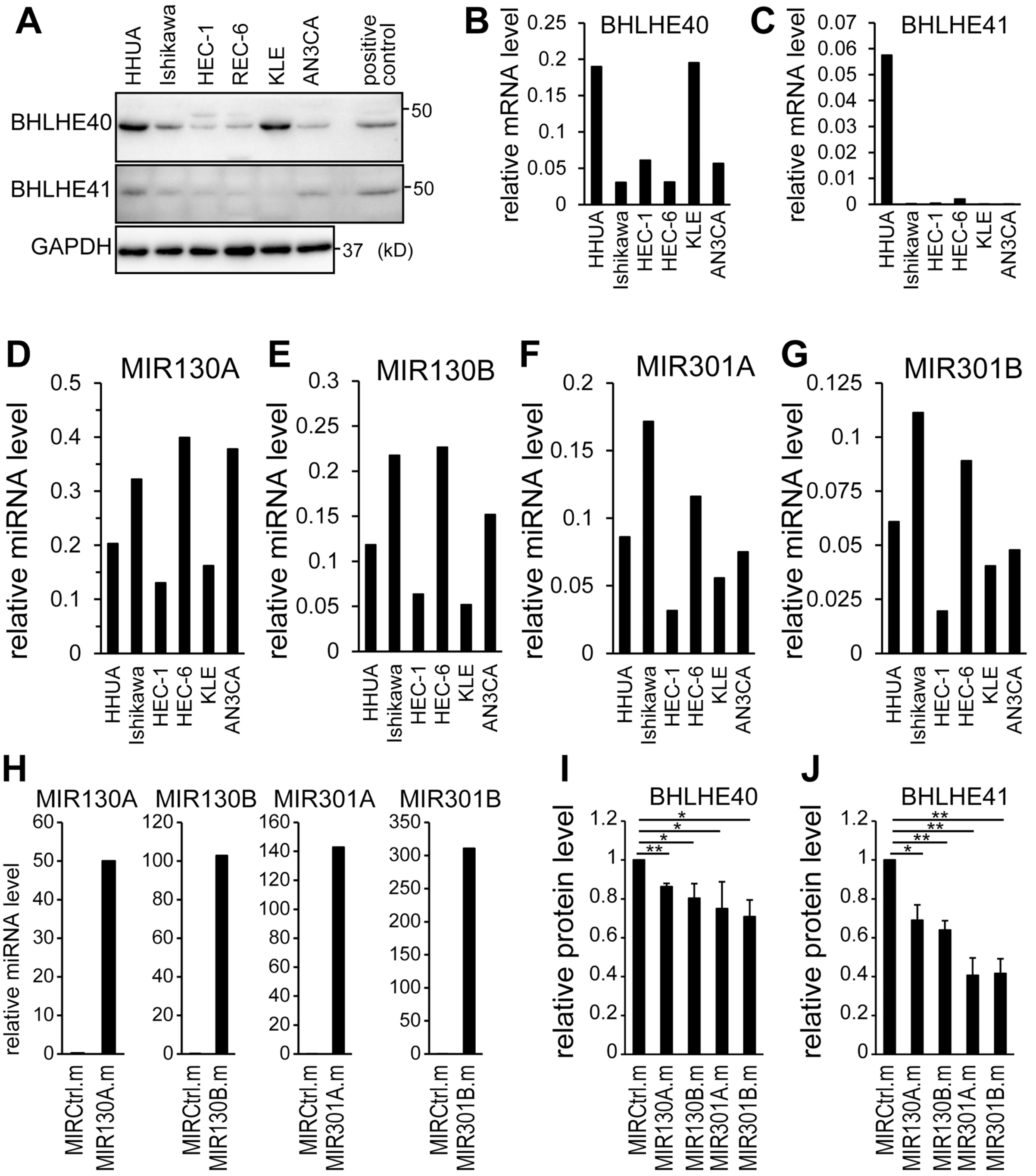

Supplementary Figure 2: (A) Expression status of BHLHE40/41 in EC cell lines analyzed by immunoblotting. Expression status of BHLHE40 **(B)** and BHLHE41 **(C)** in six EC cell lines analyzed by real-time qPCR. Expression profiles of MIR130A **(D)**, MIR130B **(E)**, MIR301A **(F)** and MIR301B **(G)** in six HEC cell lines. **(H)** Expression levels of MIR130 family members in HHUA cells transfected with their mimics at a concentration of 25 nM. Data were representative from at least three experiments. **(I, J)** Relative protein levels of BHLHE40 (I) and BHLHE41 (J) in HHUA cells transfected with mimics of MIR130 family members at a concentration of 25 nM were quantified measuring intensity of bands from three independent immunoblots. Representative blots were shown in Figure 3A. MIRCtrl.m, control microRNA mimic; MIR130A.m, MIR130A mimic; MIR130B.m, MIR130B mimic; MIR301A.m, MIR301A mimic; MIR301B.m, MIR301B mimic; *, $P < 0.05$; **, $P < 0.01$.

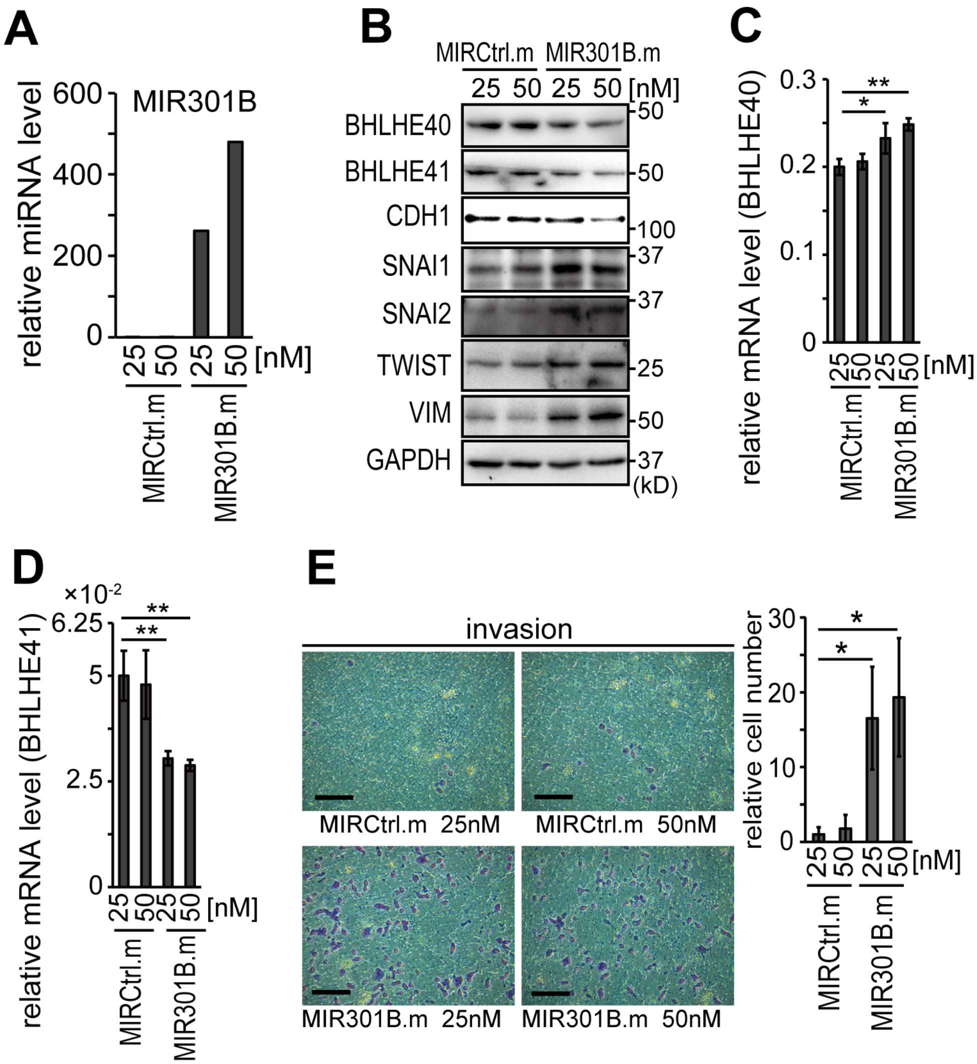

Supplementary Figure 3: (A) Expression level of MIR301B in HHUA cells transfected with control and MIR301B mimic at a concentration of 25 or 50 nM. **(B)** Protein expression of BHLHE40/41 and EMT markers in HHUA cells used in (A). mRNA levels of BHLHE40 **(C)** and BHLHE41 **(D)** in HHUA cells used in (A, B). **(E)** *In vitro* cell invasion of HHUA cells used in (A-D). The right graph shows quantification data of the results. The scale bars indicate 200 µm. Data were representative from at least three experiments. MIRCtrl.m, control microRNA mimic; MIR301B.m, MIR301B mimic; *, $P < 0.05$; **, $P < 0.01$.

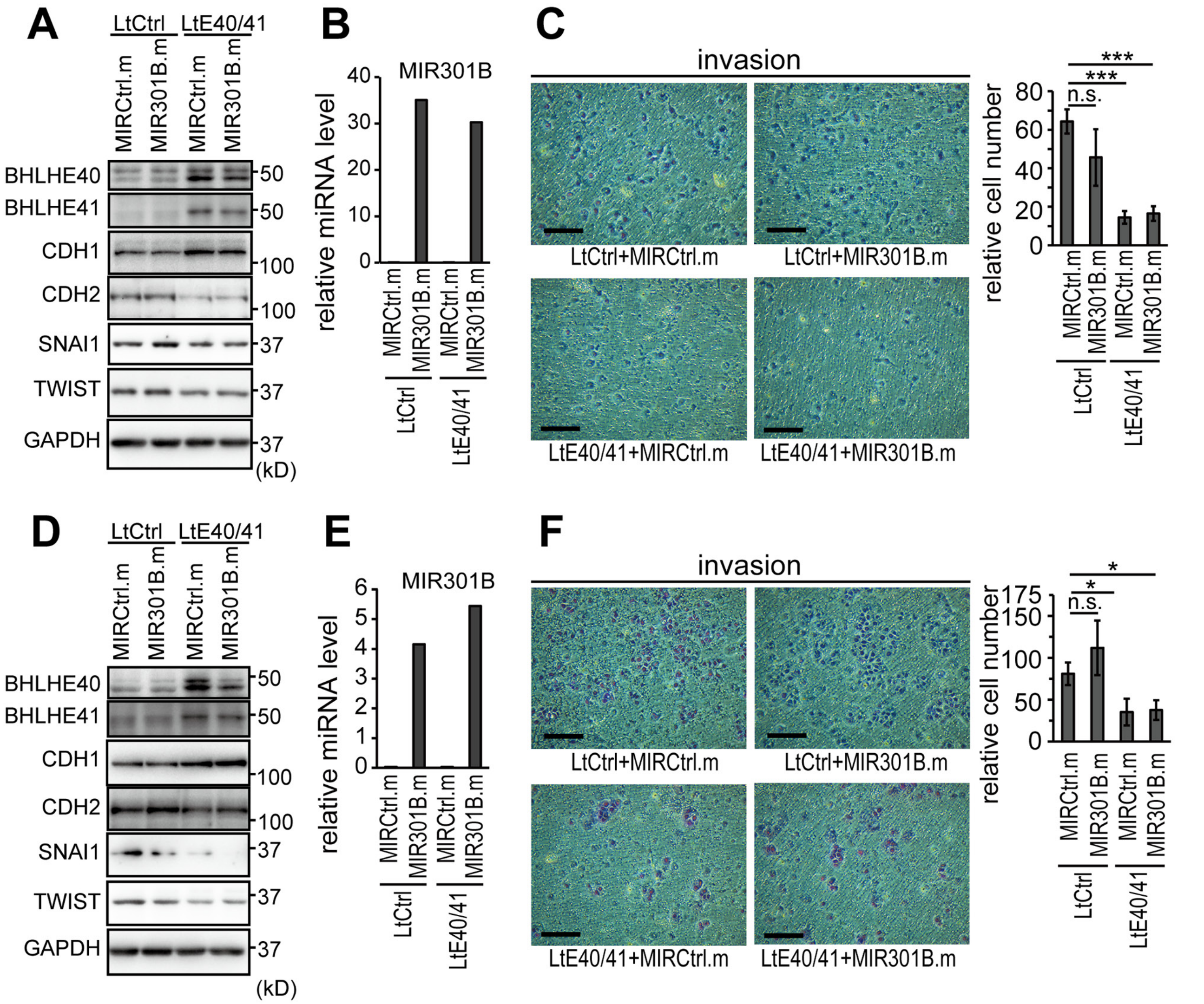

Supplementary Figure 4: (A) Protein expression of EMT markers in control or BHLHE40/41 expressing HEC-1 cells transfected with a control or MIR301B mimic. **(B)** Expression level of MIR301B in HEC-1 cells used in (A). **(C)** *In vitro* cell invasion of HEC-1 cells used in (A, B). **(D)** Protein expression of EMT markers in control or BHLHE40/41-expressing HEC-6 cells transfected with a control or MIR301B mimic. **(E)** Expression level of MIR301B in HEC-6 cells used in (D). **(F)** *In vitro* cell invasion of HEC-6 cells used in (D, E). The right graph shows quantification data of the results (C, F). The scale bars indicate 200 μm. Data were representative from at least three experiments. LtE40/41, LtBHLHE40+LtBHLHE41; MIRCtrl.m, control microRNA mimic; MIR301B.m, MIR301B mimic; n.s., not significant; *, $P < 0.05$; ***, $P < 0.001$.

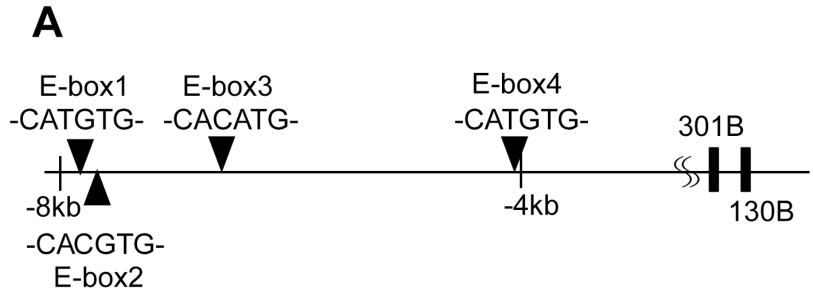

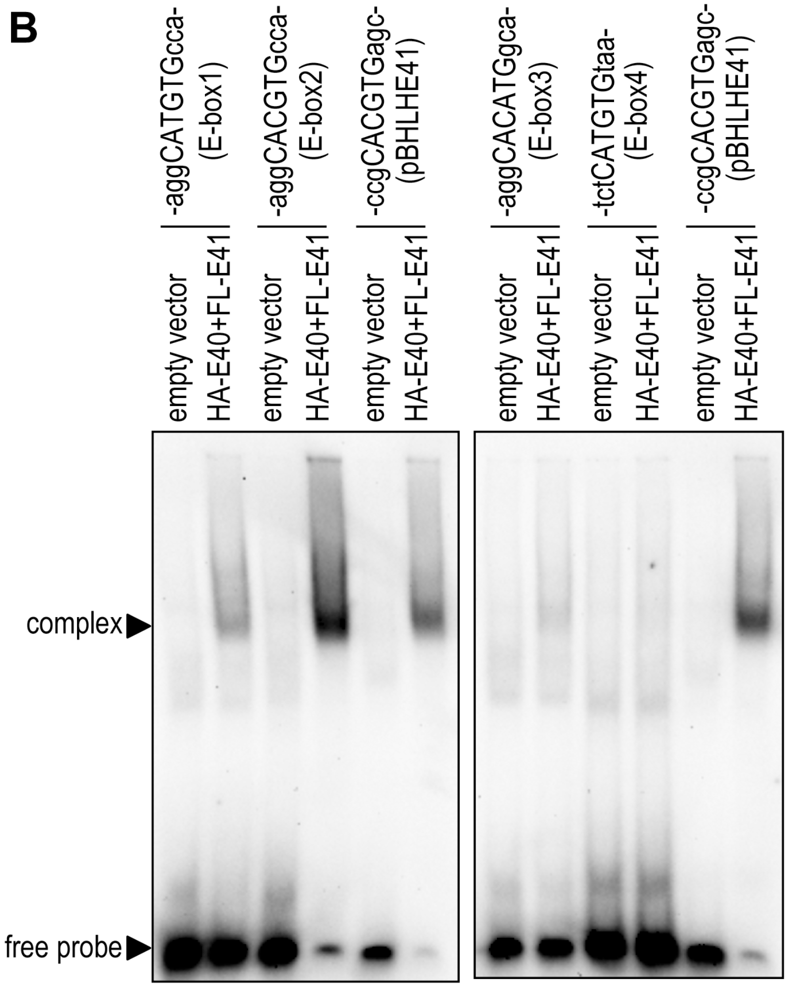

Supplementary Figure 5: Complex formation of BHLHE40/41 with candidate E-box sequences in the *MIR301B-MIR130B* promoter. (A) Schematic presentation of four candidate E-box sites in the *MIR301B-MIR130B* promoter. **(B)** EMSA assay using nuclear extract from 293T cells transfected with both HA-BHLHE40 and HA-BHLHE41. The nuclear extracts were incubated with the four labeled E-box probes (Supplementary Table 2). A canonical E-box probe from the BHLHE41 promoter was used as a positive control [1]. FL, FLAG; E40, BHLHE40; E41, BHLHE41.

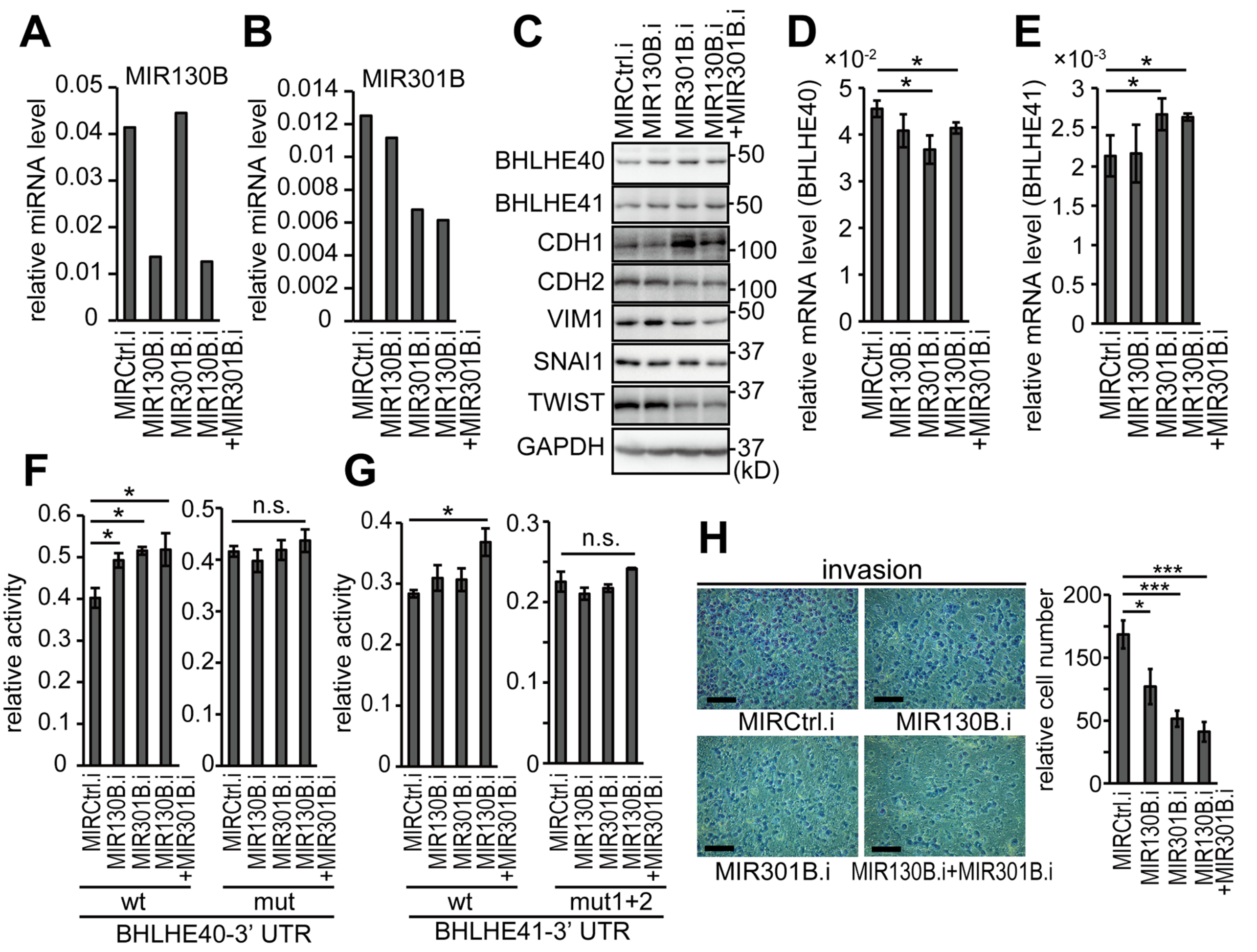

Supplementary Figure 6: Expression levels of MIR130B **(A)** and MIR301B **(B)** in HEC-1 cells transfected with their inhibitors at a concentration of 50 nM. **(C)** Protein expression of BHLHE40/41 and EMT markers in HEC-1 cells used in (A, B). mRNA levels of BHLHE40 **(D)** and BHLHE41 **(E)** in HEC-1 cells used in (A-C). The reporter activities using 3`-UTRs of BHLHE40 **(F)** and BHLHE41 **(G)** in response to inhibitors of MIR130B and MIR301B are shown. The left graphs show the results from wild-type reporters and the right graphs show the results from mutant reporters (F, G). **(H)** *In vitro* cell invasion of HEC-1 cells used in (A-E). The right graph shows quantified results data (H). Data were representative from at least three experiments. The scale bars indicate 200 µm. MIRCtrl.i, control microRNA inhibitor; MIR130B.i, MIR130B inhibitor; MIR301B.i, MIR301B inhibitor; n.s., not significant; *, $P < 0.05$; **, $P < 0.01$; ***, $P < 0.001$.

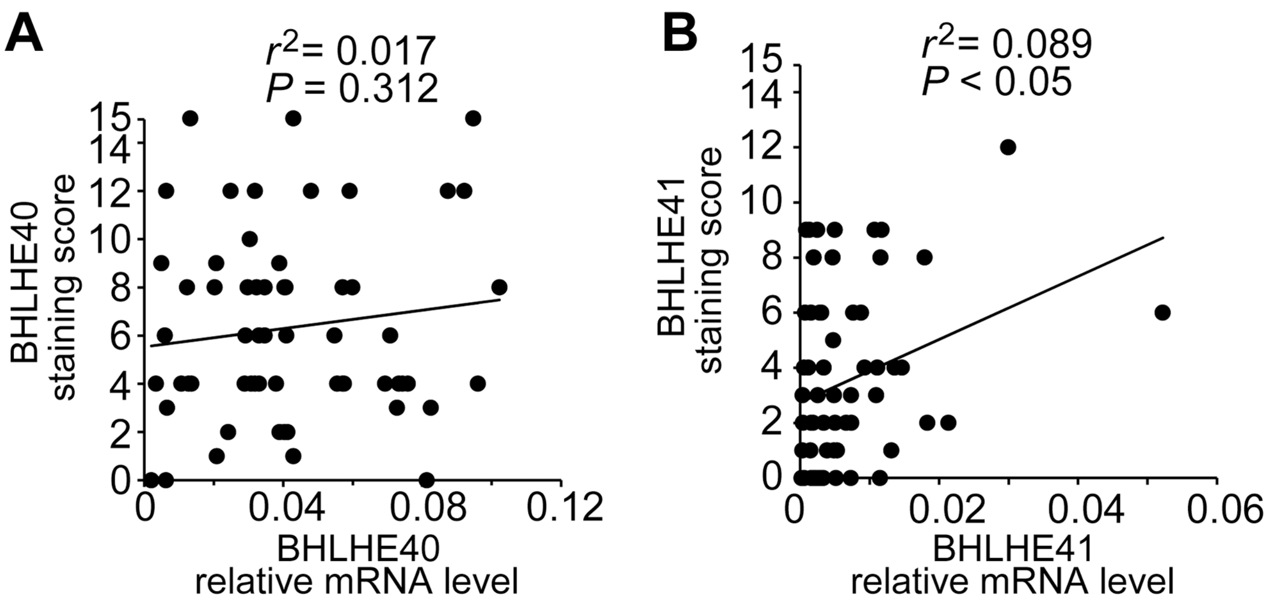

Supplementary Figure 7: The correlation between mRNA and protein levels of BHLHE40 **(A)** and BHLHE41 **(B)** from the 61 specimens was analyzed using Pearson's product-moment correlation coefficient. r-values show correlation coefficients. The significance of these relationships was determined using the *F*-test.

SUPPLEMENTARY REFERENCE

1. Li Y, Xie M, Song X, Gragen S, Sachdeva K, Wan Y, Yan B. DEC1 negatively regulates the expression of DEC2 through binding to the E-box in the proximal promoter. J Biol Chem. 2003; 278:16899–16907. https://doi.org/10.1074/jbc.M300596200. [PubMed]

Supplementary Table 1: Sequences of forward primers used to generate mutant reporter constructs

Reporter	Sense oligonucleotide
BHLHE40-3'UTR wt	+1126 AAATGCACTATGCCTGATTGCTGATCGTGTTTTAACTTTTT +1166
BHLHE40-3'UTR mut	+1126 AAAACGAGAATGCCTGATTGCTGATCGTGTTTTAACTTTTT +1166
BHLHE41-3'UTR wt1	+673 AACTTGCACTCGTTAAAGAAACACGGAGCTGG +704
BHLHE41-3'UTR mut1	+673 AACTACGAGACGTTAAAGAAACACGGAGCTGG +704
BHLHE41-3'UTR wt2	+1562 AAGTTGCACTTTTGTTACAATGCTTATGCTTGGTACAAGCT +1602
BHLHE41-3'UTR mut2	+1562 AAGTACGAGATTTGTTACAATGCTTATGCTTGGTACAAGCT +1602
pMIR130B-wtE-box	+7774 CAGGCACGTGCCACCACGCCCAGCTAAT +7747
pMIR130B-mutE-box	+7774 CAGGAAAATGCCACCACGCCCAGCTAAT +7747
pMIR130B-wtSP1BS (rs861843-C)	-70 GGCCCCGCC CCAGCCAGCCTGCAT TCCAGGTCTCAG -35
pMIR130B-wtSP1BS (rs861843-G)	-70 GGCCCGGCC CCAGCCAGCCTGCAT -47
pMIR130B-mutSP1BS	-70 AGACACACACAAGACAGCCTGCATTCCAGGTCTCAG -35

Mutated nucleotides are underlined.

Supplementary Table 2: Sequences of oligonucleotide probes

Probe	Sense oligonucleotide
pMIR301B-E-box1	-7941 GGACTACAGGCATGTGCCACCATGC -7917
pMIR301B-wtE-box2	-7780 GGATTACAGGCACGTGCCACCACG -7757
pMIR301B-mutE-box2	-7780 GGATTACAGGAAAATGCCACCACG -7757
pMIR301B-E-box3	-6667 CACCATAGGCACATGGCATCTCAG -6644
pMIR301B-E-box4	-4100 AGTGGCTCTCATGTGTAATCCCAGTA -4075
pBHLHE41-E-box	+36 CGTTCCGCACGTGAGCTGGG +55
pMIR130B-wtSP1 binding site (BS) (rs861843-C)	-77 GGGCAGAGGCCCCGCCCCAGCCAGCCTGCATT -46
pMIR130B-wtSP1BS (rs861843-G)	-77 GGGCAGAGGCCCGGCCCCAGCCAGCCTGCATT -46
pMIR130B-mutSP1BS	-46 GGGCAGAAGACACACACAAGACAGCCTGCATT -46

Altered nucleotides are underlined.

Supplementary Table 3: Primers used for qRT-PCR and ChIP-PCR analyses

Symbol	Accession No.	Forward primer	Reverse primer	Amplicon (bp)
BHLHE40, ORF	NM_003670	5’-GACCGGATTAAC GAGTGCAT-3’	5’-TGCTTTCACAT GCTTCAAGG-3’	123
BHLHE41, ORF	NM_030762	5’-GCATGAAACGAGA CGACACC-3’	5’-ATTTCAGATGT TCAGGCAGT-3’	126
BHLHE40, 3’-UTR	NM_003670	5’-GCACCGCCAGAT CCTTTCTG-3’	5’-CGGAGCTGGCT GGACACAT-3’	147
BHLHE41, 3’-UTR1	NM_030762	5’-GGATGCAGTTTC TCACGTTGC-3’	5’-GCCCAGCTCCG TGTTTCTTT-3’	92
BHLHE41, 3’-UTR2	NM_030762	5’-TCTTTTGGGGAGG ATTTGCTGA-3’	5’-CCAGCTGGTTTC TCACAGACA-3’	120
ACTB	NM_001101	5’-TTGCCGACAGGA TGCAGAAG-3’	5’-CAGCGAGGCCAG GATGGAGC-3’	122
E-box		5’- AGATTCCGCCTC CCAGGTTC -3’	5’- ACCAGCCTGGCC AACATCAT -3’	138
SP1BS		5’- CCTTCTCCATGGA AACTTGA -3’	5’- CAGGGATCTGAG ACCTGGAA -3’	120
